# Antihyperuricemia, Antioxidant, and Antibacterial Activities of *Tridax procumbens* L.

**DOI:** 10.3390/foods8010021

**Published:** 2019-01-10

**Authors:** Yusuf Andriana, Tran Dang Xuan, Tran Ngoc Quy, Truong Ngoc Minh, Truong Mai Van, Tran Duc Viet

**Affiliations:** 1 Graduate School for International Development and Cooperation, Hiroshima University, Hiroshima 739-8529, Japan; yusufandriana@yahoo.com (Y.A.); tnquy@ctu.edu.vn (T.N.Q.); minhtn689@gmail.com (T.N.M.); truongmaivan1991@gmail.com (T.M.V.); viettran1609@gmail.com (T.D.V.); 2 Development Center for Appropriate Technology, Indonesian Institute of Sciences, Jl. KS. Tubun No. 5 Subang, Jawa Barat 41213, Indonesia

**Keywords:** *Tridax procumbens*, beverage, xanthine oxidase, antioxidant, antibacterial activity

## Abstract

*Tridax procumbens* L. is a medicinal plant and used as a drink to treat bronchial catarrh, diarrhea, dysentery and liver diseases. In this study, we evaluated the potential use of *T. procumbens* to treat hyperuricemia, oxidative stress, and bacterial infection. Ethyl acetate extract of this plant was separated to different fractions by column chromatography (CC) using chloroform and methanol as eluents and subjected to xanthine oxidase (XO) inhibitory, antioxidant, and antibacterial assays. The results showed that the F_45–47_ fraction exhibited the strongest XO inhibitory activity (IC_50_ = 133.17 µg/mL), while the F_48–50_ fraction possessed maximum antioxidant activity assessed by DPPH (2,2-diphenyl-2-picrylhydrazyl) and ABTS (2,2’-azinobis (3-ethylbenzothiazoline-6-sulfonic acid) assays (IC_50_ = 0.51 and 1.04 mg/mL, respectively). In addition, the F_4–5_ fraction presented the most effective inhibition on the growth of *Escherichia coli*, *Staphylococcus aureus*, *Bacillus subtilis*, and *Proteus mirabilis*. Gas chromatography-mass spectrophotometry (GS-MS) and liquid chromatography-electrospray ionization-mass spectrophotometry (LC-ESI-MS) results revealed that fatty acids, glycerides, and flavonoids were the major compounds of the F_45–47_ fraction. Glycerides, triose sugar alcohols, and fatty acids were dominant compounds of the F_48–50_ fraction, while sterols were principal components of the F_4–5_ fraction. This study indicated that *T. procumbens* had potent inhibitory effects on XO inhibitory, antioxidant, and antibacterial activities. These biological activities may be attributed to the presence of fatty acids, flavonoids, and sterols in this plant. It is suggested that *T. procumbens* can be utilized as a healthy source to develop beverages and foods to treat antihyperuricemia, oxidative stress, and bacterial infection.

## 1. Introduction

Although *Tridax procumbens* L. (Asteraceae) was reported as a weed to invade in many crops, it has been long employed as a traditional drink to cure treat bronchial catarrh, diarrhea, dysentery [1], and liver diseases [2,3] in many countries in Africa, South and Southeast Asia. Many bioactive compounds, such as procumbetin [1], 8,3′-dihydroxy-3,7,4′-trimethoxy-6-*O*-β-d-glucopyranosyl flavone, 6,8,3′-trihydroxy-3,7,4′-trimethoxyflavone; puerarin [4], centaurein, and centaureidin, have been successfully isolated from this plant [5]. Lipid constituents of this plant, including methyl 14-oxooctadecanoate, methyl 14-oxononacosanoate, 30-methyl-28-oxodotriacont-29-en-l-oic acid, β-amyrone, β-amyrin; lupeol, and fucosterol, have been identified [6]. Furthermore, phenolic acids, including benzoic, vanilic, benzeneacetic acids, and guiacol, from this plant have been determined [7].

*T. procumbens* possesses a wide spectrum of biological activities. The ethyl acetate extract of this plant showed strong allelopathic and larvicidal activities [7,8]. In pharmaceutical activities, methanol and ethanol extracts exhibited anti-hyperglycemic [9], anti-fungal [10], anti-leshmanial [11], and hepatoprotective activities [12], while ethyl acetate extract exerted anti-inflammatory, anti-cyclooxygenase, and antioxidant activities [5]. The acetone extract of this herb obtained anticoagulant, anti-hepertic, antibacterial activities [13]. However, information on the anti-hyperuricemia property of this plant has not been yet documented.

Hyperuricemia is an abnormality high level of uric acid in the blood. Normal uric acid levels are 2.4–6.0 mg/dL for female and 3.4–7.0 mg/dL for male [14]. High level of uric acid in the blood stimulates gout, a type of inflammatory arthritis caused by the deposition of monosodium urate crystal in synovial fluid and other tissues [15]. Xanthine oxidase (XO) is the key enzyme responsible for uric acid production by catalysis hypoxanthine into xanthine, and xanthine in turn into uric acid. It plays a vital role to cause hyperuricemia and gout [16,17]. To date, only allopurinol and febuxostat have been clinically approved as XO inhibitors to treat hyperuricemia and gout disease. However, due to negative effects of these XO inhibitors, such as hepatitis, nephropathy, and allergic reaction [18], new alternative natural products prepared in safer consumption, such as beverages with enhanced therapeutic properties and less side effects, are desired.

On the other hand, interests have also increased in finding naturally occurring antioxidants and antibacterial agents for use in food or medicinal materials from *T. procumbens* [19,20]. Ethanol and methanol extracts of this plant noted to have antioxidant activity [5,21]. Aqueous, ethanol, and methanol extracts of this weed possessed antibacterial activity against *Escherecia coli* [22,23], while its ethyl acetate extract showed antibacterial activity against *Staphylococcus aureus*, *Salmonella typhi*, *Klebsiella pneumoniae*, *Escherichia coli*, and *Bacillus cereus* [20]. However, bioactive constituents that responsible for biological activities in the plant have not been well examined. Thus, the present study was conducted to examine the xanthine inhibitory, antioxidant, and antibacterial activities of *T. procumbens*. The hot water extract of this plant relevant to these biological properties has also examined in order to evaluate potent utilization of this plant as a healthy source for the development of foods and beverages.

## 2. Materials and Methods

### 2.1. Chemicals and Bacteria Strains

Folin-Ciocalteu’s phenol, potassium phosphate monobasic and dibasic, xanthine, xanthine oxidase, allopurinol, and hydrochloric acid were obtained from Sigma-Aldrich Japan K.K., Tokyo, Japan. Aluminium (III) chloride hexahydrate, 1,1-diphenyl-2-picrylhydrazyl (DPPH), sodium acetate, acetic acid, dibutyl hydroxuytoluene (BHT), gallic acid, and quercetin were purchased from Kanto Chemical Co. Inc., Tokyo, Japan. Acetone, potassium peroxodisulfate, 2,2’-azinobis (3-ethylbenzothiazoline-6-sulfonic acid) (ABTS) were obtained from Nacalai Tesque, Inc., Kyoto, Japan. Methanol (MeOH), ethyl acetate (EtOAc), ethanol, and methanol plus were obtained from Junsei Chemical Co., Ltd., Tokyo, Japan. All of the bacteria strains, including *Escherichia coli*, *Proteus mirabilis*, *Staphylococcus aureus*, and *Bacillus subtilis*, were purchased from Sigma-Aldrich (Tokyo, Japan).

### 2.2. Plant Materials

The samples of *Tridax procumbens* were collected on December 2016 in Subang, Indonesia (6°33′56.0″ S 107°44′54.9″ E). The materials were authenticated at Herbarium Bogoriense, Botany Division, Research Center for Biology, Indonesian Institute of Sciences, Indonesia. They were cleaned with tap water, air dried, cut into small pieces, dried in a tray dryer at 40 °C, and pulverized into fine powder, then stored in sealed containers at 4 °C until used. The species voucher of the plant (No. PPBC161210) was dried and sterilized before deposited at Laboratory of Plant Physiology and Biochemistry, Graduate School for International Development and Cooperation (IDEC), Hiroshima University, Higashi-Hiroshima, Japan.

### 2.3. Preparation of Plant Extract

An amount of 1.2 kg of *T. procumbens* powder was immersed in 16 L of methanol for seven days at room temperature. After filtration, the filtrate was then evaporated under vacuum at 45 °C using a rotary evaporator (SB-350-EYELA, Tokyo Rikakikai Co., Ltd., Tokyo, Japan) to obtain 62.95 g of crude extract. This crude extract was then subsequently extracted with hexane, chloroform, EtOAc, and water to produce 10.37, 14.12, 5.42, 23.54 g extracts, respectively. The EtOAc extract, the extracts active on XO inhibitory and antioxidant activities were subsequently fractionated by column chromatography.

### 2.4. Fractionation of Ethyl Acetate Extract

**Figure 1:**
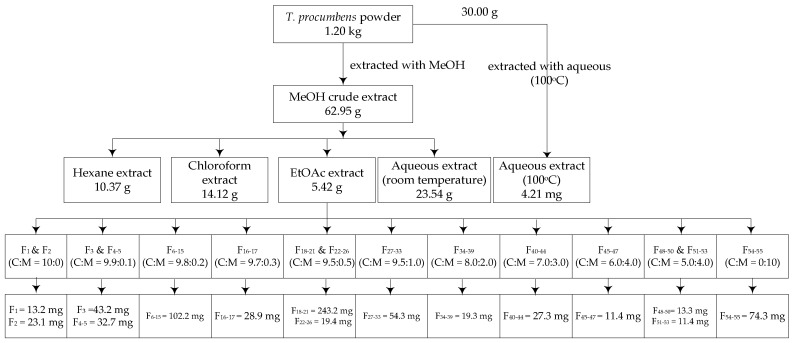
Fractionation ethyl acetate extract of *T. procumbens*. C = chloroform, M = methanol.

The EtOAc extract (5.42 g) was subjected to a normal-phase of column chromatography (20 mm diameter × 500 mm height, Climbing G2, Tokyo, Japan) over 30 g of silica gel (size Ǻ 60, 200–400 mesh particle size, Sigma-Aldrich). This process yielded 15 fractions (Figure 1) with following eluents: F_1_ and F_2_ in CHCl_3_; F_3_ and F_4–5_ in CHCl_3_: MeOH (9.9:0.1), F_6–15_ in CHCl_3_: MeOH (9.8:0.2), F_16–17_ in CHCl_3_: MeOH (9.7:0.3), F_18–21_ and F_22–36_ in CHCl_3_: MeOH (9.5:0.5), F_27–33_ in CHCl_3_: MeOH (9.0:1.0), F_34–39_ in CHCl_3_: MeOH (8.0:2.0), F_40–44_ in CHCl_3_: MeOH (7.0:3.0), F_45–47_ in CHCl_3_: MeOH (6.0:4.0), F_48–50_ and F_51–53_ in CHCl_3_: MeOH (5.0:5.0), and F_54–55_ in MeOH. All of these fractions were tested for XO inhibitory, antioxidant, and antibacterial activities. The most active fractions were analyzed by gas chromatography-mass spectrometry (GC-MS) and liquid chromatography-electrospray ionization- mass spectrometry (LC-ESI-MS) to determine their chemical components.

### 2.5. Xanthine Oxidase (XO) Inhibitory Activity

The XO inhibitory activity was assayed spectrofotometrically in vitro under aerobic condition at 290 nm based on a method reported previousy [17,24]. Briefly, a volume of 50 µL of tests solution dissolved in in phosphate buffer (pH = 7.5) contained <0.1% DMSO was mixed with 35 µL of 70 mM phosphate buffer (pH = 7.5), and 30 µL of fresh enzyme solution (0.01 units/mL in 70 mM phosphate buffer, pH = 7.5). Reaction was initiated by adding 60 µL of substrate solution (150 µM xanthine in the same buffer) after pre-incubation at 25 °C for 15 min. The assay mixture was then incubated at 25 °C for 30 min. A volume of 25 µL HCl (1 M) was added to stop the reaction and the absorbance was measured at 290 nm with a microplate reader (MultiskanTM Microplate Spectrophotometer, Thermo Fisher Scientific, Osaka, Japan). For the blank, the assay mixture was prepared in its present condition, but the enzyme solution was added after adding HCl. One unit of XO was defined as the amount of enzyme required to produce 1 µmol of uric acid/min at 25 °C. The XO inhibitory activity was expressed as the percentage inhibition of XO in the above assay system and calculated by the following formula:

(1) $$\%\;\mathrm{Inhibition}=\;\left\{\frac{\left(A-B\right)-\left(C-D\right)}{A-B}\right\}\times 100,$$

where A was the activity of the enzyme without either test extract or fraction, B was the control of A without either test extract or fraction and enzyme, C and D were the activities of either the test extract or fraction with and without XO. Allopurinol (10–100 µg/mL) was used as a positive control. The IC_50_ values were calculated from the mean values of percentage inhibition data.

### 2.6. Antioxidant Activity

#### 2.6.1. DPPH Radical Scavenging Activity

The 1,1-diphenyl-2-picryhydrazyl (DPPH) was used to evaluated radical scavenging activity as described previously [25]. The mixture assay consisted of 100 µL sample dissolved in MeOH, 50 µL of 0.2 mM DPPH solution, and 100 µL of 0.1 M acetate buffer (pH 5.5) were put in a 96-wells microplate and incubated at room temperature in the dark condition for 30 min. The absorbance was recorded at 517 nm using a microplate reader (Multiskan^TM^ Microplate Spectrophotometer, Thermo Fisher Scientific, Osaka, Japan) and butylated hydroxytoluene (BHT) (10–100 µg/mL) was used as a positive control. Percentage of inhibition was calculated according to the formula:

(2) $$\%\;\mathrm{Radical}\;\mathrm{scavenging}\;=\frac{\left({\mathrm{Abs}}_{\mathrm{control}}-{\mathrm{Abs}}_{\mathrm{sample}}\right)}{{\mathrm{Abs}}_{\mathrm{control}}}\times 100.$$

The Abs_control_ was the absorbance of reaction without sample and Abs_sample_ was the absorbance of reaction with the sample. The IC_50_ values were the concentrations required to give 50% DPPH radical scavenging activity, were also calculated.

#### 2.6.2. ABTS Radical Scavenging Activity

The 2,2′-azinobis (3-ethylbenzothiazoline-6-sulfonic acid) radical cation (ABTS) solution was used to examine the radical scavenging activity followed a method previously described [25] with slight modifications. The ABTS solution was generated by a reaction of 7 mM ABTS and 2.45 mM potassium persulfate solution after incubation at room temperature in the dark for 16 h. The mixture was then diluted with methanol to obtain an absorbance of 0.70 ± 0.05 at 734 nm. In brief, a volume of 24 µL sample dissolved in MeOH was mixed with 120 µL of ABTS solution, and the mixture was left in the dark at room temperature for 30 min. The absorbance was recorded at 734 nm using microplate a reader (Multiskan^TM^ Microplate Spectrophotometer, Thermo Fisher Scientific, Osaka, Japan). BHT standard (5–125 µg/mL) was used as a positive control. The ABTS radical scavenging activity was calculated by the following equation:

(3) $$\%\;\mathrm{Radical}\;\mathrm{scavenging}\;=\frac{\left({\mathrm{Abs}}_{\mathrm{control}}-{\mathrm{Abs}}_{\mathrm{sample}}\right)}{{\mathrm{Abs}}_{\mathrm{control}}}\times 100.$$

The Abs_control_ was the absorbance of reaction without sample and Abs_sample_ was the absorbance of reaction with the sample. The IC_50_ values were determined as the inhibitory concentration of the samples necessary to reduce the ABTS radical cation concentration by 50% and were expressed in mg/mL.

### 2.7. Antibacterial Activity

The disk diffusion method described previously [26] was used for antibacterial assay. Four bacteria strains, including *Escherichia coli*, *Proteus mirabilis*, *Staphylococcus aureus*, and *Bacillus subtilis*, were grown in Luria-Bertani (LB) broth medium by incubated at 37 °C for 24 h. The final population was standardized to be 1.45 × 10^6^ CFU/mL (*E. coli*), 2.87 × 10^6^ CFU/mL (*P. mirabilis*), 1.29 × 10^6^ CFU/mL (*S. aureus*), and 1.63× 10^6^ CFU/mL (*B. subtilis*). A volume of 100 µL of bacteria culture was covered evenly on agar—LB broth medium in a Petri dish (diameter = 9 cm). Afterward, a volume of 20 µL of sample dissolved in MeOH was applied into filter paper (6 mm diameter) and placed on the surface of LB agar plates. After 24 h incubation at 37 °C, the inhibition zones were measured. Streptomycin and ampicillin were used as the positive controls in this experiment. The concentrations of the samples ranged from 1.25 to 40 mg/mL (40, 30, 25, 20, 10, 5, 2.5, 1.5, 1.25 mg/mL). The lowest concentration that inhibited the visible bacterial growth was determined as minimal inhibitory concentration (MIC). Streptomycin and ampicillin (1.25, 0.625, 0.313, 0.156, 0.078, 0.039, 0.0195, 0.0097, 0.0048, 0.0024, 0.0012, 0.0006 mg/mL) were used as positive control in this experiment. Subsequently, MeOH was used as a negative control.

### 2.8. Determination of Total Phenolic Contents

The total phenolic contents of the samples were measured by the Folin Ciocalteu (FC) reagent following a method reported previously [27] with some modifications. Briefly, a volume of 20 µL of either sample solution (1.0 mg/mL), or gallic acid standard solution (5–25 µg/mL) was pipetted into separate wells of a 96-well microplate. Then a volume of 100 µL of the FC reagent (10% *v*/*v* in water) was added to each well, thoroughly mixed, and an aliquot of 80 µL sodium carbonate (5% *w*/*v* in water) was then added. The reaction was carried out for 30 min at room temperature. The absorbance was read at 765 nm using a microplate reader (Multiskan^TM^ Microplate Spectrophotometer, Thermo Fisher Scientific, Osaka, Japan). The total phenolic contents were expressed as mg gallic acid equivalent (GAE) per gram of extract or fraction (*r*^2^ = 0.996).

### 2.9. Determination of Total Flavonoid Contents

The total flavonoid contents were assessed by a colorimetric assay as described previously [28], with some modifications. Briefly, a volume of either 100 µL sample (1 mg/mL) or quercetin standard (5–25 µg/mL) was mixed with 100 µL aluminum (III) chloride hexahydrate (2% *w*/*v* in water) in a 96-well microplate. After a 15-min incubation at room temperature, the absorbance of the reaction mixture was measured at 430 nm using a microplate reader (Multiskan^TM^ Microplate Spectrophotometer, Thermo Fisher Scientific, Osaka, Japan). The total flavonoid contents were expressed as mg quercetin equivalent (QE) per gram of extract or fraction (*r*^2^ = 0.999).

### 2.10. Identification of Chemical Constituents by Gas Chromatography-Mass Spectrometry (GC-MS)

A volume of 1 µL of sample was injected into a GC-MS system (JMS-T100 GCV, JEOL Ltd., Tokyo, Japan). The column was DB-5MS with 30 m in length, 0.25 mm internal diameter, and 0.25 µm in thickness (Agilent Technologies, J&W Scientific Products, Folsom, CA, USA). Helium was chosen as the carrier gas, and the split ratio was 5.0/1.0. The operating condition of GC oven temperature was maintained as follows: The initial temperature = 50 °C without hold time, the programmed rate = 10 °C/min up to a final temperature of 300 °C with 20 min for hold time. The injector and detector temperatures were set at 300 °C and 320 °C, respectively. The mass range scanned from 29–800 amu. The control of the GC-MS system and the data peak processing were carried out using the JEOL’s GC-MS Mass Center System version 2.65a software (JEOL Ltd., Tokyo, Japan).

### 2.11. Liquid Chromatography-Electrospray Ionization-Mass Spectrometry (LC-ESI-MS) Analysis

Identification of the most active fraction was performed by LC-ESI-MS system (Thermo Fisher Scientific^TM^, LTQ XL^TM^, Ion Trap Mass Spectrometer, Tokyo, Japan). The column for LC system was JASCO J-Pak Symphonia C18 (250 mm × 4.6 mm × 5 μm). The mobile phase was 0.1% formic acid in water (solvent A) and 0.1% formic acid in acetonitrile (solvent B). The proportion of solvents A:B was 30:70 and the flowrate was 0.4 mL/min. Operation time of this analysis was 30 min and the volume of sample injection was 5 µL. For ESI-MS conditions, the ionization method was electrospray (ESI). The flow rate of sheath gas was 60, while auxiliary gas was 20 arbitrary units of sheath gas. The spray voltage was 4.5 kV. The measurements were performed in the positive mode. For positive polarity, fourier transform mass spectrometry (FTMS)/orbitrap with 60,000 resolution and scan range 100–1000 *m*/*z* was applied for mass analyzer. For negative polarity, ion trap mass spectrometer (ITMS)/linear ion trap with scan range 115–1000 *m*/*z* was employed [29]. Peak processing was conducted using Thermo Xcalibur Qual Browser software (Thermoscientific^TM^, Tokyo, Japan) equipped with NIST MS Library.

### 2.12. Statistical Analysis

The statistical analysis was performed by one-way ANOVA using Minitab^®^ 16.2.3 (copyright© 2012 Minitab Inc., Philadelphia, PA, USA). The results were presented as means ± standard deviation (SD) values. Differences among treatments, controls and standard data were considered significant at *p* < 0.05 using Fisher’s test.

## 3. Results

### 3.1. XO Inhibitory and Antioxidant Activities, Total Phenolic and Flavonoid Contents of T. procumbens Extracts

The XO inhibitory and antioxidant activities of *T. procumbens* extracts were shown in Table 1. The results indicated that EtOAc extract exhibited the strongest inhibitory activity in both XO and antioxidant assays. At 0.1 µg/mL dose, the EtOAc extract inhibited 19.44% of XO, while the others showed negligible inhibition. In antioxidant activity, the EtOAc extract also possessed maximum inhibition on antioxidant activity. The IC_50_ values of EtOAc extracts of both DPPH and ABTS radical scavenging assays were the lowest (0.13 and 0.45 mg/mL, respectively). It was observed that XO inhibitory and antioxidant activity of *T. procumbens* extracts were proportional with total phenolic and flavonoids contents.

### 3.2. Antibacterial Activity of T. procumbens Extracts

In the antibacterial activity assay, different extracts of *T. procumbens* were tested against four bacteria strain, including *E. coli*, *P. mirabilis*, *S. auereus*, *B. subtilis*. The EtOAc extract had the strongest inhibitory effects on all bacteria strain (minimum inhibitory activity = 25–10 mg/mL). (Table 2). Hexane extract gave inhibition on *E. coli* and *S. auereus*, while chloroform extract only inhibited *S. auereus*.

### 3.3. XO Inhibitory and Antioxidant Activities of T. procumbens Fractions

All of the 15 fractions separated from the EtOAc extract of *T. procumbens* were tested for XO inhibitory and antioxidant activities (Table 3). In the XO inhibitory activity, the F_45–47_ fraction showed the most potential inhibition (IC_50_ = 133.17 µg/mL) followed by the F_27–33_ and F_22–26_ fractions (IC_50_ = 150.71 and 188.04 µg/mL, respectively). Other fractions possessed trivial inhibitory activities which were not considerable enough to calculate IC_50_ values. Compared with allopurinol, all fractions presented lower inhibitory levels. In antioxidant activity, most of the fractions possessed both DPPH and ABTS radical scavenging activities. The F_48–50_ fraction showed the most effective antioxidant potential (IC_50_ DPPH and ABTS were 0.51 and 1.04 mg/mL, respectively).

### 3.4. Antibacterial Activity of T. procumbens Fractions

Antibacterial activity of *T. procumbens* was assayed on Gram positive (*E. coli* and *P. mirabilis*) and negative (*S. auereus* and *B. subtilis*) bacteria. The inhibitory effects of *T. procumbens* fractions on tested bacteria were illustrated in Table 4. The inhibition levels of tested fractions on four bacteria strain were varied. The F_4–5_ fraction was the best candidate to inhibit the growth of all tested bacteria (MIC = 15–25 mg/mL). All fractions showed lower inhibitory levels than ampicillin and streptomycin which were used as the positive controls. Ampicillin and streptomycin gave MIC values of 0.0012–0.039 and 0.156 mg/mL respectively.

### 3.5. Total Phenolic Contents (TPC) and Total Flavonoid Contents (TFC) of T. procumbens Fractions

**Figure 2:**
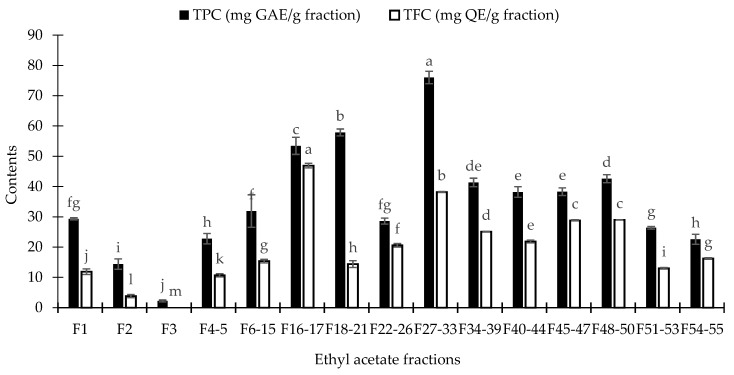
Total phenolics (TPC) and flavonoids (TFC) contents of EtOAc fraction. Data are presented as means ± standard deviation (SD). Means with different letters on bars are significantly different by Fisher’s test (*p* < 0.05).

The TPC and TFC of all fractions were illustrated in Figure 2. The F_27–33_ fraction showed the highest phenolic contents, while F_16–17_ fraction exhibited the maximal flavonoids contents. Nevertheless, neither F_27–33_ nor F_16–17_ fractions provided maximum inhibition on XO inhibitory, antioxidant, and antibacterial activities. Besides phenolic and flavonoid compounds, constituents belonging to other chemical classes probably related to these inhibitory activities.

### 3.6. Compounds Identification by Gas Chromatography-Mass Spectrometry (GC-MS) and Liquid Chromatography-Electrospray Ionization-Mass Spectrometry (LC-ESI-MS)

The identification of the most active fraction in XO inhibitory (F_45–47_ fraction), antioxidant (F_48–50_ fraction), and antibacterial (F_4–5_ fraction) activities was conducted by using GC-MS and LC-ESI-MS (Supplementary Materials Figures S1–S15). The results were summarized in Table 4. Chemicals belonging to fatty acids, glycerides, and flavonoid groups were detected in the F_48–50_ fraction. In this fraction, the major compound was n-hexadecanoid acid (16.56%) followed by 2-monopalmitin (11.98%), and centaureidin (2.63%) (Table 5). For antioxidant activity, five compoundsa were identified in F_48–50_ fraction, of which 2-monopalmitin was the most dominant compound, followed by glycerin (11.04%) and methyl palmitate (5.67%). In the antibacterial activity, four compounds in the chemical classes of sterols and fatty acids were detected in F_4–5_ fraction. In details, sterols (ergost-5-en-3-ol, (3β)-, stigmasterol, and β-sitosterol) were noted as the principal compounds (52.02%).

## 4. Discussion

In this study, we screened bioactive compounds from *T. procumbens* using extracting solvents with different polarities by column chromatography. The EtOAc extract of this plant gave the most inhibitory effects in XO, antibacterial, and antioxidant assays. This extract was subsequently selected and fractionated by column chromatography eluting by chloroform and methanol (10:0 to 0:10 *v*/*v*) to yield 15 fractions. The levels of biological activities as mentioned above of each fraction were then evaluated in vitro. It was found that *T. procumbens* fractions showed potent XO inhibitory antioxidant, and antibacterial activities. To date, XO inhibitors, such as flavonoids [30], deoxyadenosines for example cordycepin [31], lanostanoids [32], and phenolics [33], have been reported. In this study, we detected n-hexadecanoid acid, 2-monopalmitin, and centaureidin in the F_45–47_ fraction (Table 4) involved in XO inhibitory activity.

Centaureidin is one type of flavonoid compounds identified from *T. procumbens* [5]. In line with this study, Nagao et al., [30] reported that various dietary flavonoids to have inhibitory activity on XO. From chemical structure and binding affinities of flavonoids acting as XO inhibitors, Lin et al., [34] reported that generally, hydrophobic interaction was essential in the binding of flavonoids to XO inhibition. The planar structure and double bond C_2_ = C_3_ of flavonoids are advantageous for XO suppressive potential [34]. In this study, due to the inhibitory effects of the F_45–47_ fraction was potent on XO, the interaction of fatty acids, glycerides, and flavonoids responsible XO inhibition were also needed to clarify.

Regarding antioxidant activity, the F_48–50_ fraction showed the highest radical scavenging activities both in DPPH and ABTS assays. Of which, 2-monopalmitin, glycerin, and methyl palmitate were the dominant compounds detected. Similar to this result, Mohadjerani et al. [35] reported that fatty acids and their derivatives have been reported to have antioxidant activity. Methyl palmitate found in this fraction (Table 4) may also play a role in the antioxidant activity of the plant.

In antibacterial activity, the F_4–5_ fraction presented the maximal inhibitory effects on Gram positive (*E. coli* and *P. mirabilis*) and negative (*S. auereus* and *B. subtilis*) bacteria. The GC-MS and ESI-MS results revealed that stigmasterol, β-sitosterol, and n-hexadecanoid acid were the principal compounds of this fraction. In a previous report, Sharma [36], noted that stigmasterol and β-sitosterol had a wide spectrum of antibacterial activity. These sterols showed effective inhibition on *S. aureus* and *S. albus* (Gram positive), and *E. coli* and *Pseudonomas pyocyeanea* (Gram negative). In addition, Desbois and Smith [37], reported that n-hexadecanoid acid inhibited both Gram positive and negative bacteria. The insertion of fatty acid into the bacterial inner membrane increased the permeability of the membrane, causes internal contents to leak from the cell, and induces growth inhibition or even death [38]. It has been revealed that bioactive compounds from medicinal plants appeared as promising sources for natural beverages and foods [39,40,41,42,43]. Observations of this study highlighted that *T. procumbens* possessed potent constituents and may be served as a healthy source to develop healthy drinks and foods, especially in developing countries, where this plant is abundantly available.

## 5. Conclusions

Gout is a problematic issue of health in both developed and developing countries, of which utilization of natural products with high effectiveness and less undesirable effects to treat this problematic disease is required. In this study, we found that *T. procumbens* was exhibited strong inhibitory effects on XO, antioxidant and growth of four bacteria strains *E. coli*, *P. mirabilis*, *S. aureus*, and *B. subtilis*. The GC-MS and LC-ESI-MS results revealed that fatty acids, glycerides, and flavonoids may contribute to XO inhibitory activity, while sterols and fatty acids may play a role in the antibacterial property. In antioxidant activity, glycerides, triose sugar alcohols, terpenes, and fatty acids may be active on the radical scavenging inhibitory assay. Findings of this study suggested that *T. procumbens* is a promising source to develop beverages and foods to treat hyperuricemia, oxidative stress, and bacterial infection.

## Figures and Tables

**Table 1:** Xanthine oxidase (XO) inhibitory, antioxidant activity, total phenolic, and flavonoids contents of *T. procumbens* extracts.

Extracts	XO Inhibitory Activity (%) at 0.1 mg/mL	Radical Scavenging Activities		TPC (mg GAE/g Extract)	TFC (mg QE/g Extract)
		IC_50_ DPPH (mg/mL)	IC_50_ ABTS (mg/mL)		
Hexane	-	>5.00	3.05 ± 0.13 ^a^	19.24 ± 0.95 ^b^	12.01 ± 0.15 ^b^
Chloroform	-	1.40 ± 0.16 ^a^	2.11 ± 0.11 ^b^	2.67 ± 0.22 ^c^	1.23 ± 0.09 ^d^
EtOAc	19.44 ± 1.47 ^a^	0.13 ± 0.06 ^c^	0.45 ± 0.01 ^d^	99.14 ± 2.42 ^a^	38.47 ± 0.21 ^a^
Aqueous	-	0.88 ± 0.05 ^b^	1.26 ± 0.01 ^c^	18.38 ± 0.54 ^b^	2.06 ± 0.05 ^c^
Hot Aqueous (100 °C)	7.21 ± 0.88 ^b^	0.95 ± 0.02 ^b^	1.38 ± 0.01 ^c^	17.16 ± 0.97 ^b^	1.91 ± 0.07 ^c^

Data presented means ± standard deviation (SD). Values in the same column followed by similar letters are not significantly different by Fisher’s test (*p* < 0.05), - = no inhibition. EtOAc = ethyl acetate. TPC = total phenolics contents. TFC = total flavonoids contents.

**Table 2:** Antibacterial activity in term of minimal inhibitory concentration (MIC) values of *T. procumbens* extracts.

Extracts	Minimum Inhibitory Concentration (mg/mL)			
	*E. coli*	*P. mirabilis*	*S. aureus*	*B. subtilis*
Hexane	30	-	30	-
Chloroform	-	-	30	-
EtOAc	30	30	30	25
Aqueous	-	-	-	-
Ampicillin	0.0098	0.0391	0.0012	0.0195
Streptomycin	0.156	0.156	0.156	0.156

- = no inhibition.

**Table 3:** XO inhibitory and antioxidant activities of *T. procumbens* fractions.

Fractions	XO Inhibitory Activity		Radical Scavenging Activities	
	XO Inhibition (%) at 100 µg/mL	IC_50_ XO Inhibition (µg/mL)	IC_50_ DPPH (mg/mL)	IC_50_ ABTS (mg/mL)
F_1_	3.99 ± 1.92 ^g^	-	1.01 ± 0.08 ^d^	1.99 ± 0.06 ^d^
F_2_	11.35 ± 1.61 ^fg^	-	-	8.44 ± 0.39 ^a^
F_3_	-	-	-	2.52 ± 0.23 ^c^
F_4-5_	-	-	3.23 ± 0.53 ^a^	4.94 ± 0.45 ^b^
F_6-15_	-	-	1.74 ± 0.16 ^c^	1.84 ± 0.17 ^d^
F_16–17_	-	-	1.96 ± 0.44 ^bc^	1.23 ± 0.01 ^fgh^
F_18–21_	-	-	1.08 ± 0.12 ^d^	1.08 ± 0.03 ^gh^
F_22–26_	33.72 ± 0.69 ^bc^	188.04 ± 13.99 ^a^	2.29 ± 0.06 ^b^	1.48 ± 0.08 ^ef^
F_27–33_	37.11 ± 6.24 ^b^	150.71 ± 2.65 ^b^	2.92 ± 0.09 ^a^	1.19 ± 0.05 ^fgh^
F_34–39_	22.55 ± 5.42 ^de^	-	1.79 ± 0.11 ^c^	1.35 ± 0.05 ^efg^
F_40–44_	15.83 ± 1.46 ^ef^	-	1.99± 0.02 ^bc^	1.24 ± 0.01 ^fgh^
F_45–47_	40.40 ± 6.57 ^b^	133.17 ± 18.84 ^b^	2.06 ± 0.15 ^bc^	1.22 ± 0.03 ^fgh^
F_48–50_	-	-	0.51 ± 0.06 ^e^	1.04 ± 0.04 ^h^
F_51–53_	11.77 ± 1.47 ^f^	-	0.54 ± 0.03 ^e^	1.07 ± 0.03 ^gh^
F_54–55_	26.24 ± 6.35 ^cd^	-	0.82 ± 0.02 ^de^	1.53 ± 0.06 ^e^
Allopurinol *	90.21 ± 6.19 ^a^	4.85 ± 2.18 ^c^	n.d.	n.d.
BHT **	n.d.	n.d.	0.009 ± 0.001 ^f^	0.045 ± 0.014 ^f^

Data presented means ± standard deviation (SD). Values in a column with different letter are significantly different by Fisher’s test (*p* < 0.05). n.d. = not determined, - = no inhibition or not calculated. * = positive control of XO inhibitory activity. ** = positive control of antioxidant activity.

**Table 4:** Antibacterial activity in term of MIC values of EtOAc fractions isolated from *T. procumbens*.

Fractions	Minimum Inhibitory Concentration (mg/mL)			
	*E. coli*	*P. mirabilis*	*S. aureus*	*B. subtilis*
F_1_	-	25	30	-
F_2_	-	25	30	-
F_3_	-	25	25	-
F_4–5_	20	20	15	25
F_6–15_	25	-	15	-
F_16–17_	-	25	25	30
F_18–21_	25	-	-	-
F_22–26_	25	-	30	30
F_27–33_	-	-	-	40
F_34–39_	20	-	30	-
F_40–44_	-	-	-	-
F_45–47_	25	-	-	-
F_48–50_	25	20	20	-
F_51–53_	-	-	15	-
F_54–55_	-	-	30	30
MeOH	-	-	-	-
Ampicillin	0.0098	0.039	0.0012	0.0195
Streptomycin	0.156	0.156	0.156	0.156

- = no inhibition.

**Table 5:** Identification of the most active fraction by GC-MS and LC-ESI-MS.

No	Compounds	Formula	Weight	Chemical Classification	GC-MS		LC-ESI-MS	Fractions
					Rt (min)	Area (%)	[M+H]^+^ (*m*/*z*)	
1	n-Hexadecanoic acid	C_16_H_32_O_2_	256.24	fatty acid	17.10	16.56	257.24	F_4–5_ (antibacterial acticity)
2	Ergost-5-en-3-ol, (3β)-	C_28_H_48_O	400.37	sterol	27.49	5.56	401.37	
3	Stigmasterol	C_29_H_48_O	412.37	sterol	27.77	31.89	413.37	
4	β-Sitosterol	C_29_H_50_O	414.39	sterol	28.44	14.57	415.39	
6	n-Hexadecanoic acid	C_16_H_32_O_2_	256.24	fatty acid	17.10	16.56	257.24	F_45–47_ (XO inhibitory activity)
7	2-Monopalmitin	C_19_H_38_O_4_	330.28	glyceride	21.92	11.98	331.38	
8	Centaureidin	C_18_H_16_O_8_	360.31	flavonoid	27.60	2.63	361.09	
9	Dihydroxyacetone	C_3_H_6_O_3_	90.03	trioses	3.63	3.95	91.03	F_48–50_ (antioxidant activity)
10	Glycerin	C_3_H_8_O_3_	92.05	triose sugar alcohol	4.59	11.04	93.05	
11	2-Pentadecanone, 6,10,14-trimethyl-	C_18_H_36_O	268.28	terpene	15.90	3.42	269.28	
12	Methyl palmitate	C_17_H_34_O_2_	270.26	fatty acid	16.75	5.67	271.26	
13	2-Monopalmitin	C_19_H_38_O_4_	330.28	glyceride	21.92	14.28	331.38	

Rt = retention time. PA = peak area. GC-MS = gas chromatography-mass spectrometry. LC-ESI-MS = liquid chromatography-electrospray ionization-mass spectrometry.

## Supplementary Materials

The following are available online at http://www.mdpi.com/2304-8158/8/1/21/s1. Figure S1. GC-MS chromatogram of F_4–5_ fraction, Figure S2. ESI-MS spectra of n-hexadecanoid acid in F_4–5_ fraction, Figure S3. ESI-MS spectra of Ergost-5-en-3-ol, (3β)- in F_4–5_ fraction, Figure S4. ESI-MS spectra of Stigmasterol in F_4–5_ fraction, Figure S5. ESI-MS spectra of β-Sitosterol in F_4–5_ fraction, Figure S6. GC-MS chromatogram of F_45–47_ fraction, Figure S7. ESI-MS spectra of n-hexadecanoid acid in F_45–47_ fraction, Figure S8. ESI-MS spectra of 2-Monopalmitin in F_45–47_ fraction, Figure S9. ESI-MS spectra of Centaureidin in F_45–47_ fraction, Figure S10. GC-MS chromatogram F_48–50_ fraction, Figure S11. ESI-MS spectra Dihydroxyacetone in F_48–50_ fraction, Figure S12. ESI-MS spectra of Glycerin in F_48–50_, Figure S13. ESI-MS spectra of 2-Pentadecanone, 6,10,14-trimethyl- in F_48–50_ fraction, Figure S14. ESI-MS spectra of Methyl palmitate in F_48–50_ fraction, Figure S15. ESI-MS spectra of 2-Monopalmitin in F_48–50_ fraction.
