# Supplementary material for: Antihyperuricemia, Antioxidant, and Antibacterial Activities of Tridax procumbens L
Source: Foods. 2019 Jan 10;8(1):21. doi: 10.3390/foods8010021 (PMC6352254; doi:10.3390/foods8010021)
Supplement: Supplementary file 1 [file foods-08-00021-s001.pdf]

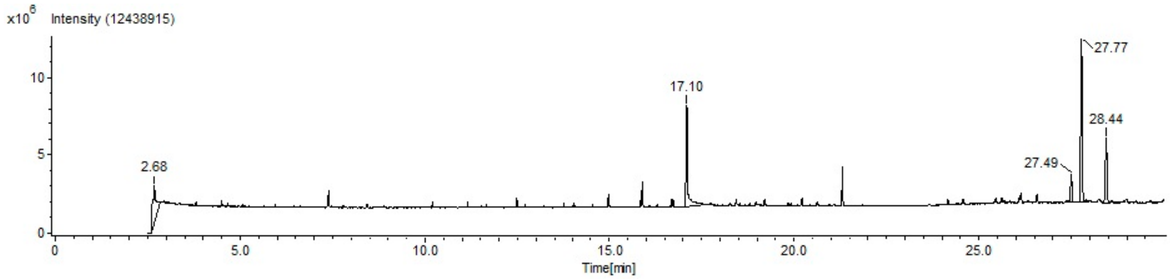

Figure S1. GC-MS chromatogram F4-5 fraction.

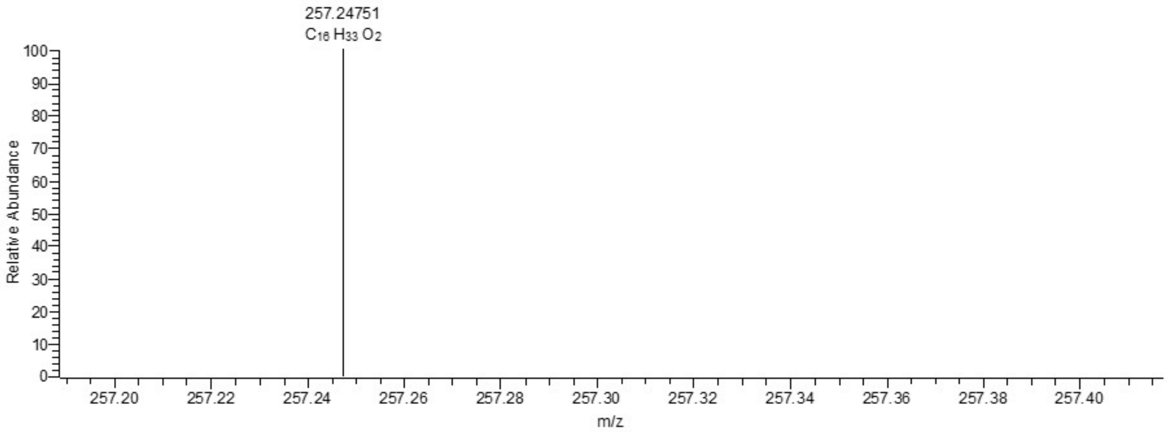

Figure S2. ESI-MS spectra of n-hexadecanoid acid in F4-5 fraction.

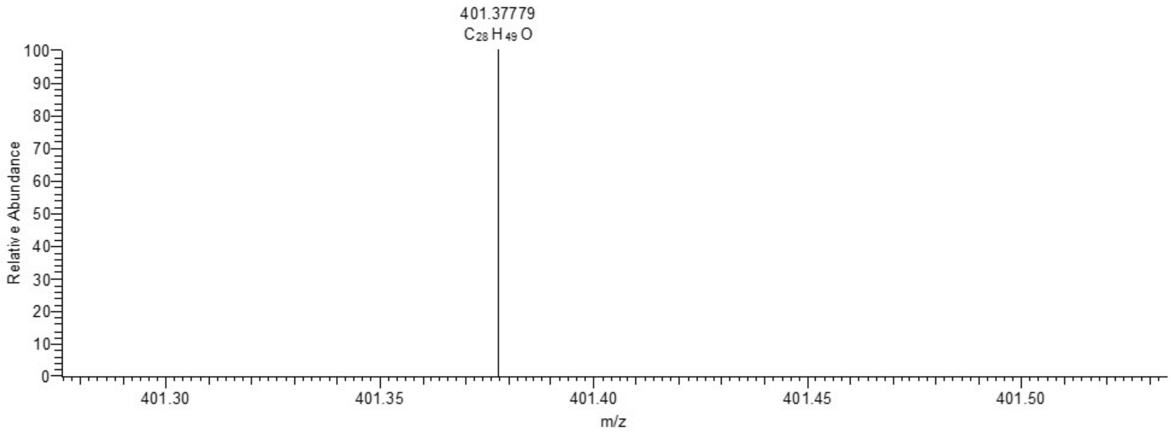

Figure S3. ESI-MS spectra of Ergost-5-en-3-ol, (3β)- in F4-5 fraction.

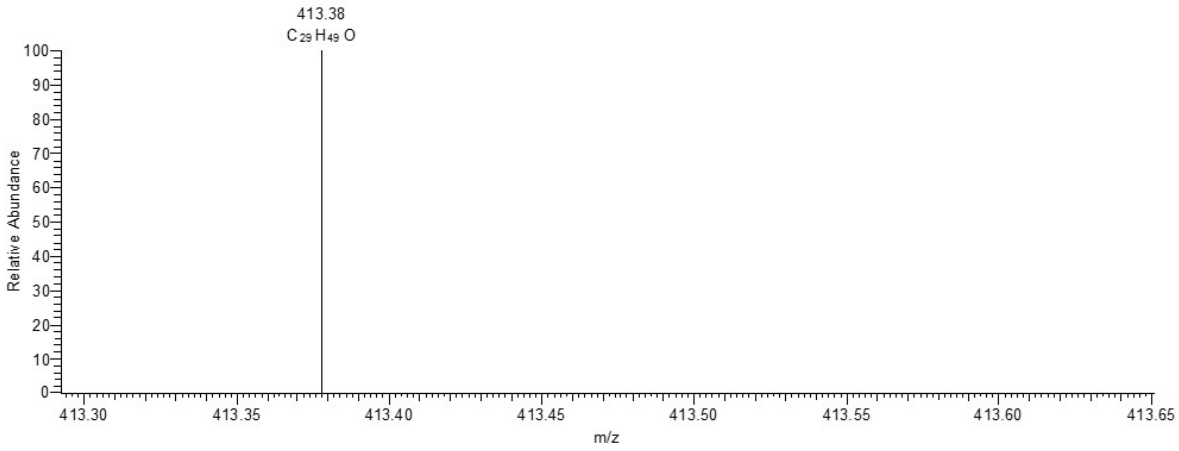

Figure S4. ESI-MS spectra of Stigmasterol in F4-5 fraction.

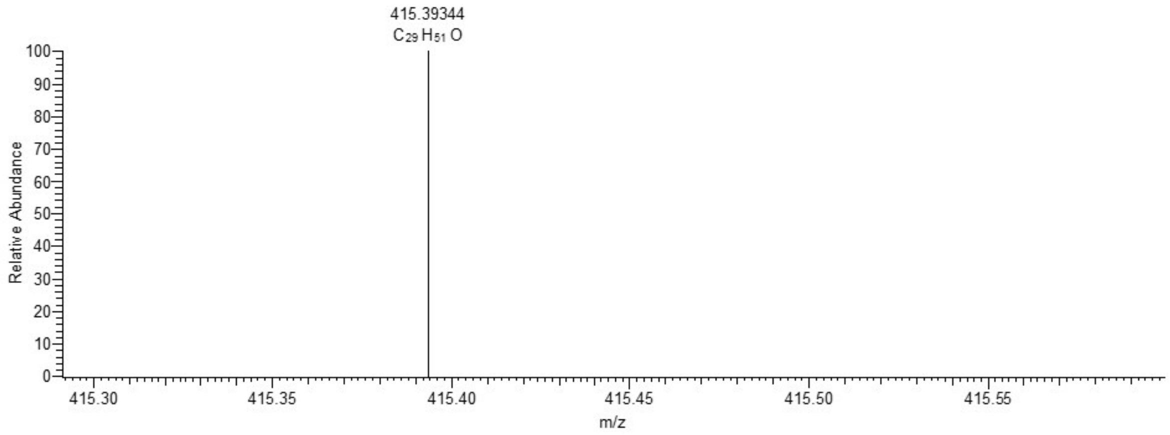

Figure S5. ESI-MS spectra of β-Sitosterol in F4-5 fraction.

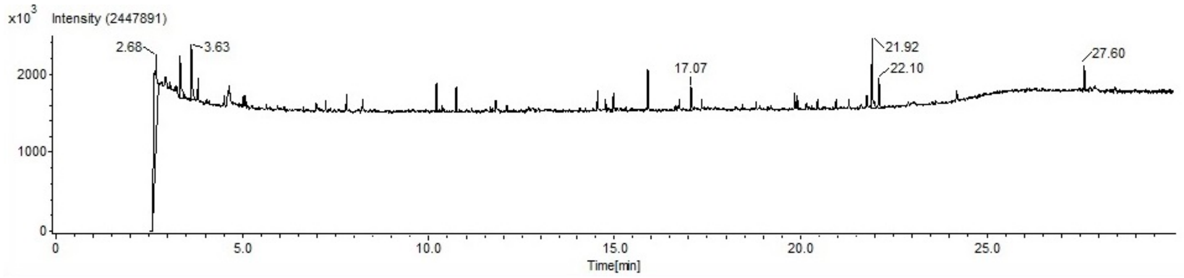

Figure S6. GC-MS chromatogram F45-47 fraction.

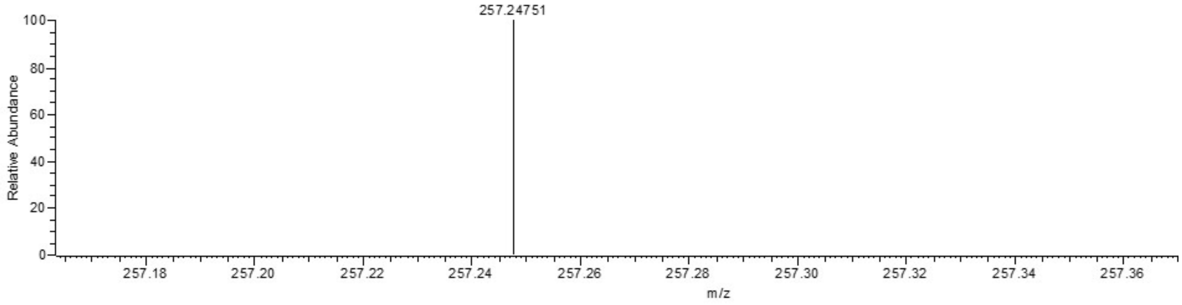

Figure S7. ESI-MS spectra of n-hexadecanoid acid in F45-47 fraction.

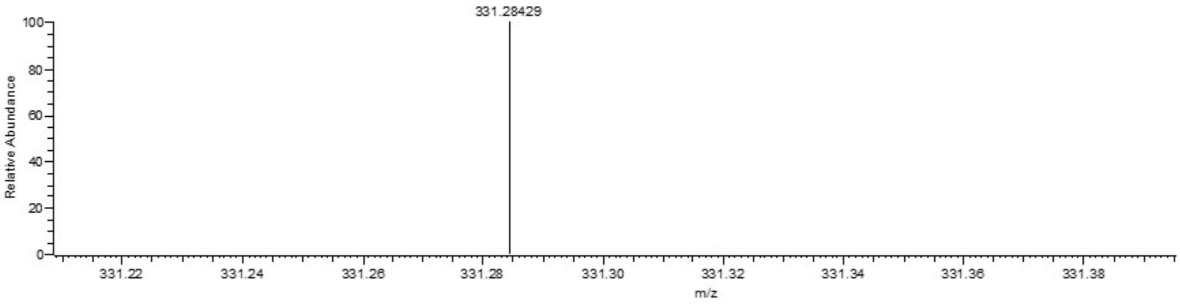

Figure S8. ESI-MS spectra of 2-Monopalmitin in F45-47 fraction.

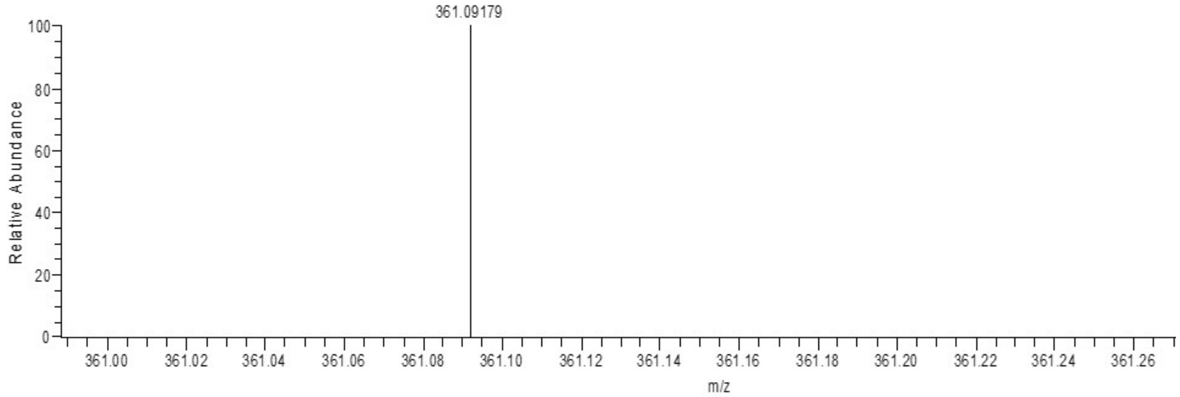

Figure S9. ESI-MS spectra of Centaudeirin in F45-47 fraction.

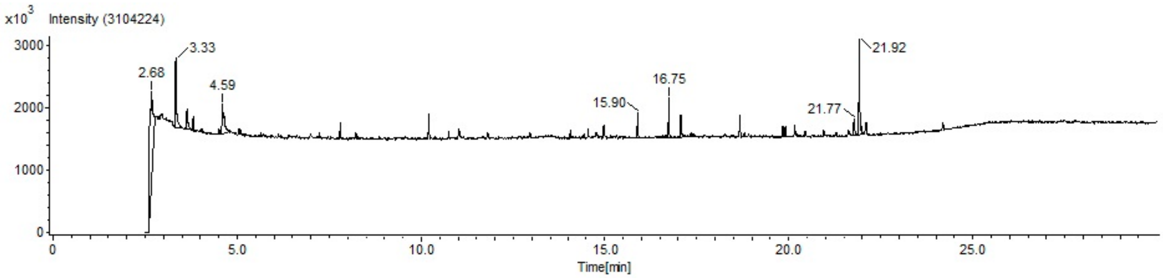

Figure S10. GC-MS chromatogram F48-50 fraction.

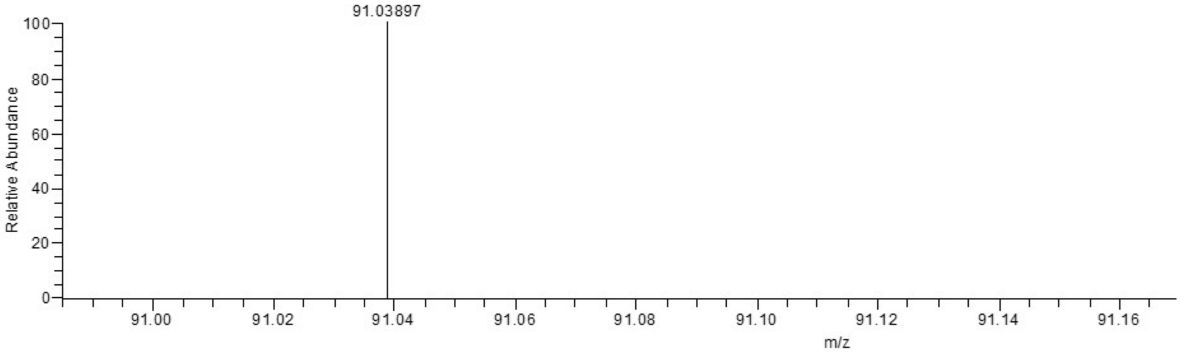

Figure S11. ESI-MS spectra Dihydroxyacetone in F48-50 fraction.

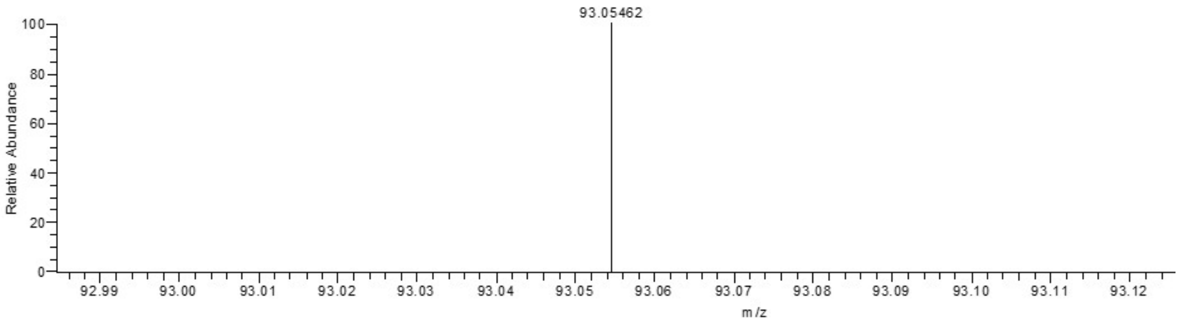

Figure S12. ESI-MS spectra of Glycerin in F48-50.

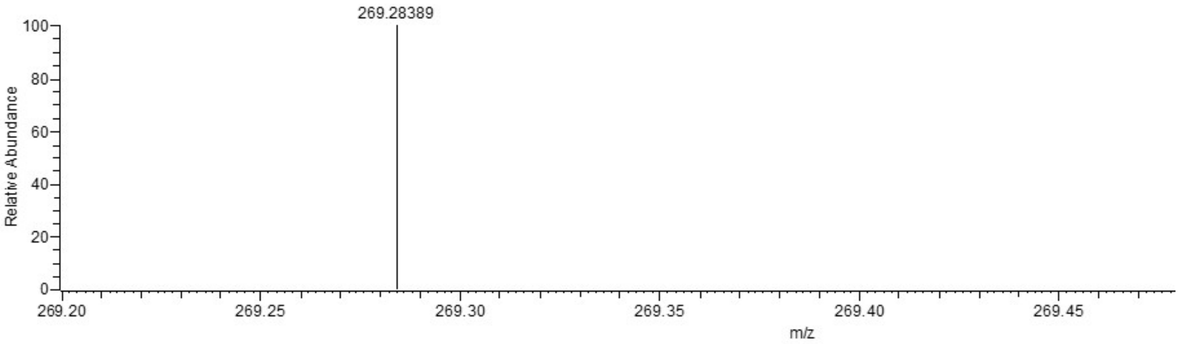

Figure S13. ESI-MS spectra of 2-Pentadecanone, 6,10,14-trimethyl- in F48-50 fraction.

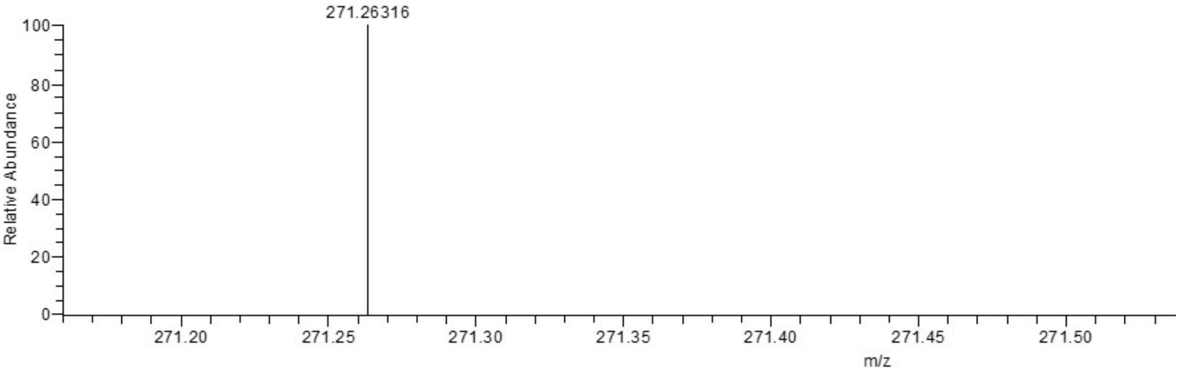

Figure S14. ESI-MS spectra of Methyl palmitate in F48-50 fraction.

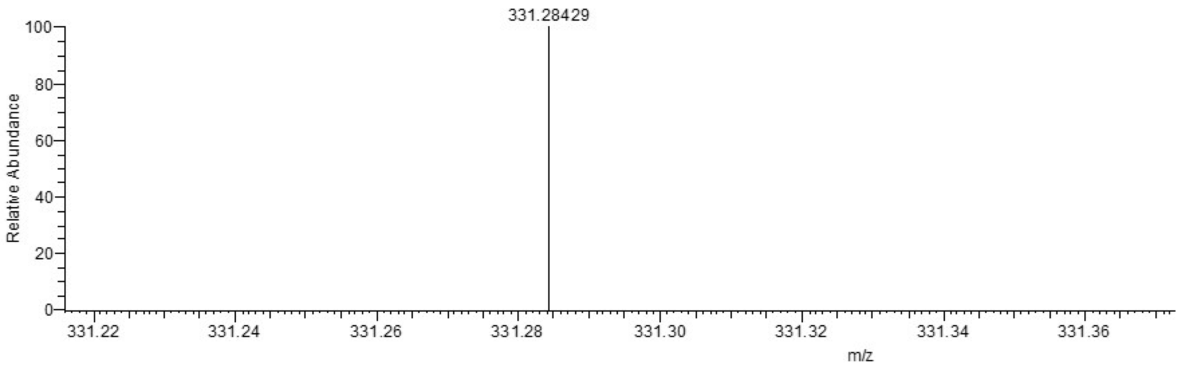

Figure S15. ESI-MS spectra of 2-Monopalmitin in F48-50 fraction.
